# The lncRNA GAS5-encoded micropeptide facilitates influenza virus replication through modulation of the Wnt/β-catenin signaling pathway

**DOI:** 10.1016/j.crmicr.2026.100559

**Published:** 2026-01-27

**Authors:** Xinni Zhou, Xiaojuan Chi, Benqun Peng, Ming Gao, Ning Li, Lu Liu, Jie Zeng, Yuxin Li, Yuzhang Chen, Song Wang

**Affiliations:** a Key Laboratory of Animal Pathogen Infection and Immunology of Fujian Province, College of Animal Sciences, Fujian Agriculture and Forestry University, Fuzhou 350002, China; b Key Laboratory of Fujian-Taiwan Animal Pathogen Biology, College of Animal Sciences, Fujian Agriculture and Forestry University, Fuzhou 350002, China; c Joint Laboratory of Animal Pathogen Prevention and Control of Fujian-Nepal, College of Animal Sciences, Fujian Agriculture and Forestry University, Fuzhou 350002, China

**Keywords:** Long non-coding RNA, GAS5, Influenza A virus, Micropeptide, Wnt/β-catenin signaling pathway

## Abstract

Long non-coding RNAs (lncRNAs) have been implicated in various cellular processes, including the regulation of gene expression and cellular response to viral infections. Herein, our RNA-seq analysis revealed a significant increase in the expression of an annotated lncRNA, GAS5, following influenza A virus (IAV) infection. Stimulation of cells with type I interferon, type III interferon or IL-6 can also result in upregulation of GAS5 expression. Additionally, overexpression of GAS5 promoted IAV replication, while knockdown of GAS5 decreased viral titers. Notably, we identified a novel 50-amino acid micropeptide encoded by GAS5, named GAS5-P50, through ribosome profiling and mass spectrometry analysis. It was found that overexpression of GAS5-P50 alone could facilitate the replication of IAV; conversely, frameshift mutation-mediated silencing of GAS5-P50 diminished the capacity of GAS5 to promote IAV replication, implying that GAS5-P50 is essential for GAS5-mediated enhancement of viral replication. Moreover, synthetic GAS5-P50 was demonstrated to boost IAV propagation both *in vitro* and *in vivo*. Mechanistically, GAS5-P50 interacted with NOTUM, a negative regulator of Wnt signaling, leading to enhanced Wnt/β-catenin pathway activation, which facilitated viral replication. These findings uncover a previously unrecognized function of GAS5 as a proviral lncRNA that encodes a functional micropeptide, which modulates host Wnt/β-catenin signaling to support IAV infection. Our study not only expands the understanding of lncRNA-encoded micropeptides in viral pathogenesis but also highlights GAS5-P50 as a potential target for antiviral intervention.

## Highlights

• Influenza virus infection significantly induces the expression of lncRNA GAS5 via type I/III interferons and IL-6 signal pathways.
• LncRNA GAS5 can encode a critical micropeptide GAS5-P50 which facilitates influenza virus replication.
• GAS5-P50 interacts with NOTUM to promote the activation of the Wnt/β-catenin pathway.

## Graphical abstract

**Figure:**
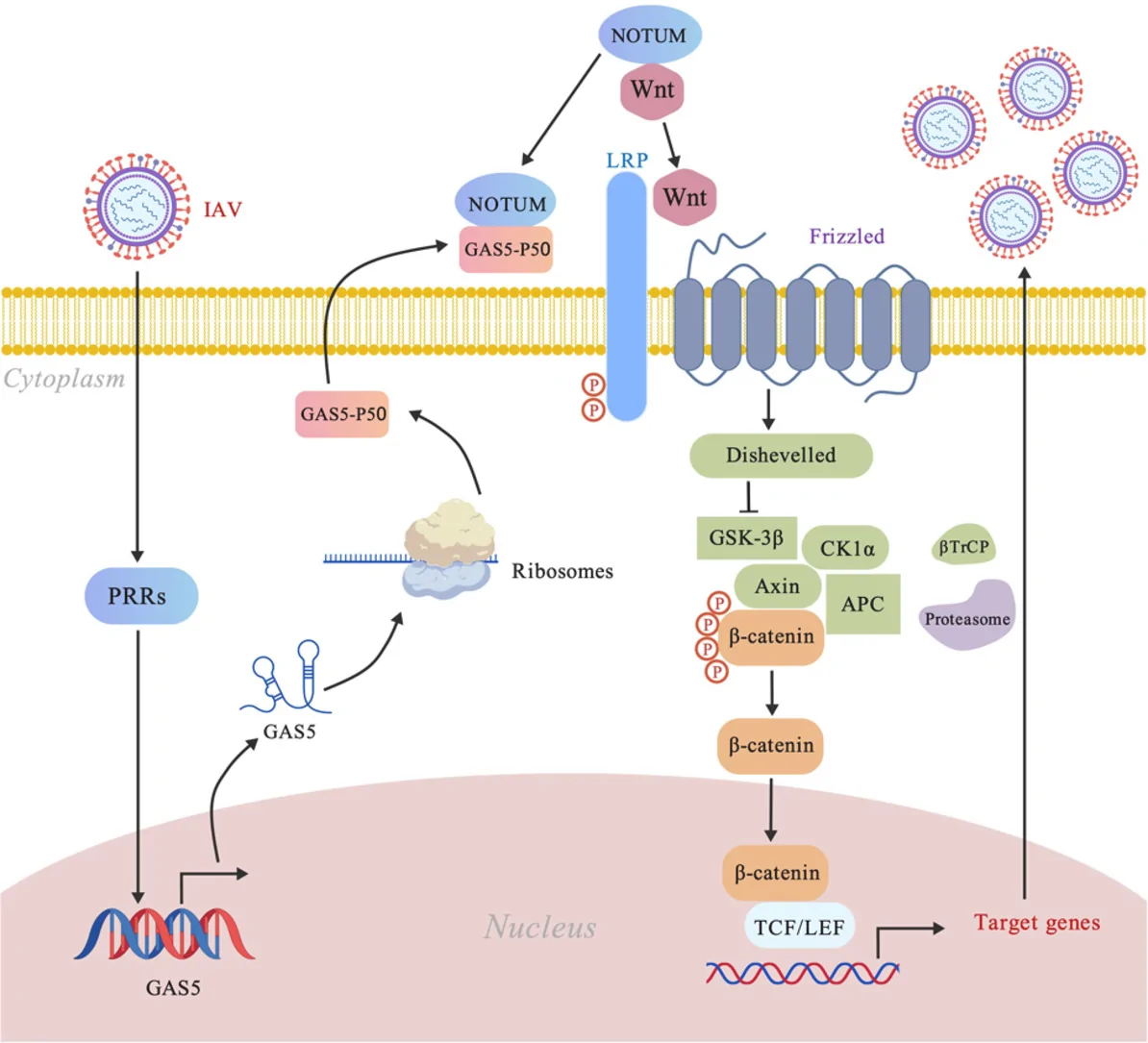

## 1 Introduction

Influenza A virus (IAV), an enveloped negative-sense RNA virus belonging to the *Orthomyxoviridae* family, continues to pose a substantial threat to global public health (Shi et al., 2023a). Characterized by high mutation rates and the capacity for genetic reassortment, IAV undergoes continuous antigenic drift, facilitating immune evasion and contributing to recurrent seasonal epidemics as well as occasional pandemics associated with significant morbidity and mortality worldwide (Long et al., 2019; Maurer et al., 2025). Although advancements in antiviral therapies and vaccines have been made, the rapid evolutionary dynamics of IAV present persistent challenges for effective disease control. This underscores the critical need to elucidate host-virus interactions to uncover novel therapeutic strategies.

Host factors that modulate IAV replication represent promising targets for antiviral therapy. Among these, long non-coding RNAs (lncRNAs), once considered non-functional, have emerged as key regulators of cellular and viral processes (Chen and Kim, 2024; Ma et al., 2022; Mattick et al., 2023; Statello et al., 2021). Our previous work has identified several lncRNAs, including NRAV, GAPLINC, lncRNA-155, RDUR, LINC02574, and THRIL, that play essential roles in IAV infection (Chen et al., 2024, 2025, 2021; Ouyang et al., 2014; Rai et al., 2022; Zhang et al., 2023). These molecules exert either proviral or antiviral effects, primarily through the regulation of host innate immune responses.

Growth arrest-specific transcript 5 (GAS5), a well-known tumor-suppressive lncRNA, plays critical roles in cellular proliferation, apoptosis, and metabolic regulation (Kino et al., 2010; Larrasa-Alonso et al., 2021; Renganathan et al., 2014; Sang et al., 2021; Schneider et al., 1988; Wang et al., 2018). Initially identified for its upregulation during growth arrest, GAS5 functions as a competing endogenous RNA (ceRNA) by sponging microRNAs or interacting with transcriptional activator to modulate gene expression (Chanda et al., 2025; Guo et al., 2015; Liu et al., 2015). Accumulating evidences indicate that GAS5 is downregulated in multiple cancers, including breast, lung, and colorectal cancers, where its loss promotes tumor progression (Mourtada-Maarabouni et al., 2009; Ni et al., 2019; Yang et al., 2021). Moreover, GAS5 has been implicated in cellular stress, bone diseases, drug resistance and immune responses (Ahmad et al., 2020; Fang et al., 2020; Li et al., 2020; Xu et al., 2025), underscoring its extensive functional importance.

Recently, emerging research has revealed that certain lncRNAs harbor small open reading frames (sORFs) capable of encoding functional micropeptides or small proteins (Jaunbocus et al., 2025; Poliseno et al., 2024). Advances in ribosome profiling (Ribo-seq) and peptidomics have enabled the identification of numerous such sORF-derived peptides (Hellinger et al., 2023; Ingolia et al., 2019), which participate in diverse biological processes, including cell signaling, gene regulation, and stress responses. For example, MLN, a skeletal muscle-specific micropeptide hidden within a presumed noncoding RNA, inhibits the SERCA calcium pump, impairing calcium uptake into the sarcoplasmic reticulum and modulating muscle relaxation (Anderson et al., 2015). SMIMP, a tumor-promoting microprotein encoded by a non-canonical ORF in ELFN1-AS1, binds SMC1A to epigenetically repress tumor suppressors, driving cancer progression (Zheng et al., 2023). P155, a 17-amino acid micropeptide from lncRNA MIR155HG, modulates antigen presentation in dendritic cells via HSC70 interaction, ameliorating inflammatory diseases in murine models (Niu et al., 2020). In our prior study, we found that PESP, a small protein encoded by lncRNA PCBP1-AS1, enhances IAV replication by promoting autophagy via ATG7 upregulation. PESP stabilizes itself by binding HSP90AA1, further amplifying viral production (Chi et al., 2024). The discovery of lncRNA-encoded micropeptides or small proteins has expanded our understanding of genomic coding potential, introducing new dimensions to cellular signaling networks. However, despite their biological significance, the roles of most lncRNA-derived peptides in IAV infection remain poorly characterized, warranting further investigation.

In this study, we identified a novel 50-amino acid micropeptide which we named GAS5-P50. It is encoded by GAS5, an annotated lncRNA upregulated during IAV infection and in response to type I/III IFNs or IL-6 stimulation. Functional studies revealed that both GAS5 and GAS5-P50 enhance viral replication, whereas disruption of the micropeptide-coding sequence completely abrogates this proviral effect. Mechanistically, GAS5-P50 interacts with NOTUM, a key suppressor of Wnt signaling, thereby activating the pathway to facilitate IAV propagation. Our findings provide novel insights into the regulatory network of lncRNA-encoded micropeptides in influenza virus pathogenesis and highlight their potential as targets for antiviral strategies.

## 2 Materials and methods

### 2.1 Cells and viruses

A549 (human type II alveolar epithelial cells), 293T (human embryonic kidney cells) and MDCK (Madin-Darby canine kidney cells) were obtained from the American Type Culture Collection (ATCC). The cells were cultured in Dulbecco’s Modified Eagle’s Medium (DMEM; Gibco, NY, USA) supplemented with 10% fetal bovine serum (FBS; Gibco) and 100 U/mL penicillin-streptomycin (Beyotime Biotechnology, Shanghai, China), and maintained at 37°C in a humidified atmosphere containing 5% CO_2_.

Influenza viruses strains, including A/PR/8/34 (H1N1) (PR8), A/WSN/33 (H1N1) (WSN), A/California/04/2009 (H1N1) (CA04), A/Chicken/Fujian/MQ01/2015 (H9N2), and a swine-origin H3N2 strain, along with Sendai virus (SeV), were propagated in specific-pathogen-free embryonated chicken eggs, whereas pseudorabies virus (PRV) was cultured in PK15 cells as previously described (Chi et al., 2024). For *in vitro* infections, cells were inoculated with each virus at the specified multiplicity of infection (MOI) in serum-free medium. After one hour of adsorption at 37°C, cells were washed twice with phosphate-buffered saline (PBS) and maintained in appropriate media: DMEM containing 2 μg/mL trypsin for influenza viruses and SeV, and DMEM supplemented with 2% FBS for PRV infections. For *in vivo* studies, 6–8 week-old female C57BL/6 J mice were anesthetized and intranasally inoculated with 5 × 10^4^ plaque-forming units (PFU) of influenza virus in 50 μL PBS. After infection for the indicated time, mice were euthanized, and lung tissues were collected for further experiments and analysis.

### 2.2 Plasmids and reagents

For plasmid construction, GAS5 and GAS5mut were cloned into the pNL-GFP vector following GFP excision. The GAS5mut was generated by introducing a two-nucleotide (AA) insertion to create a frameshift mutation specifically targeting the sORF embedded within the GAS5 sequence. GAS5-P50 was cloned into a modified pNL-GFP vector, in which the GFP start codon ATG is mutated to ATT (GFPmut), and GAS5-P50 was fused in frame to the N-terminus of the GFPmut ORF (sORF-GFPmut). The start codon of the GAS5 sORF in the sORF-GFPmut plasmid was mutated to ATT, yielding sORFmut-GFPmut. GAS5-P50 was also cloned into pLVX3 and pCAGGS vector with a 3 × Flag tag at the N-terminus and C-terminus, respectively. NOTUM was cloned into pcDNA3.1(-) vector with an HA tag at the C-terminus.

LPS was purchased from Sigma (MO, USA). IFN-β and IFN-λ1 were purchased from Peprotech (London, UK), and poly(I:C) was obtained from Invivogen (CA, USA). Wnt3a and IWP-2 were purchased from MedChemExpress (New Jersey, USA). Anti-Flag was purchased from Sigma (MO, USA). Anti-NOTUM, anti-GFP, anti-HA were purchased from Proteintech (Wuhan, China). Anti-β-actin was purchased from TransGen Biotech (Beijing, China). Anti-IAV PB2 was purchased from GeneTex (CA, USA), and anti-IAV NP was obtained as described previously (Wang et al., 2014). The peptide (PYGQLCPQGRMRIATEVLKSKPNSSHWHTGIRQKAGS) derived from GAS5-P50 was synthesized to a purity of 97%, with subsequent endotoxin removal performed by GL Biochem (Shanghai, China). Endotoxin was < 0.1 EU mg^-^¹ (LAL assay), and a scrambled peptide of identical composition or PBS served as negative controls. Peptides were dissolved in PBS as a 20 mM stock solution and stored at −80°C.

### 2.3 RNA-seq and Ribo-seq

RNA-seq and Ribo-seq were performed as described previously (Chi et al., 2024). Briefly, for RNA-seq, total RNA from PR8-infected A549 cells was extracted, followed by RNA integrity verification. Stranded cDNA libraries were prepared and sequenced on an Illumina Novaseq platform. For Ribo-seq, cultured cells were treated with cycloheximide (50 mg/mL) to arrest ribosomes, followed by lysis and nuclease digestion (RNase I) to generate ribosome-protected fragments (RPFs). After the isolation and purification of RPFs, cDNA libraries were constructed and sequenced on an Illumina Hiseq 4000 platform. The RNA-seq and Ribo-Seq data have been deposited in the GEO public database under the accession numbers GSE211357 and GSE252920, respectively.

### 2.4 RT-PCR and RT-qPCR

Total RNA was extracted using TRNzol reagent (TIANGEN, Beijing, China) following the manufacturer's protocol. cDNA was synthesized by a HiScript III 1st Strand cDNA Synthesis Kit (Vazyme, Nanjing, China). Then, the cDNA was used for PCR or quantitative PCR (qPCR) by Taq DNA polymerase (GenStar, Beijing, China) or SYBR Green Master Mix (Vazyme, Nanjing, China). GAPDH or β-actin served as the endogenous control for normalization, and relative gene expression was calculated using DDCT method by LightCycler system (Roche, Switzerland). Primer sequences used in this study are listed in Table S1.

### 2.5 Gene knockdown and overexpression

For gene knockdown, cells were transfected with target-specific or scrambled control siRNAs (RiboBio, Guangzhou, China) using Lipofectamine 3000 reagent (Invitrogen, CA, USA), followed by incubation for 48 h before analysis. Short hairpin RNA (shRNA)-based knockdown cell lines were generated by infection of A549 cells with lentiviruses expressing specific shRNAs in pSIH-H1-GFP vector as described previously (Liu et al., 2021). The specific sequences for siRNA and shRNA oligonucleotides used in this study are listed in Table S1. For gene overexpression, 293T cells were co-transfected with the lentiviral vector containing the gene of interest and the packaging plasmids using Lipo8000™ reagent (Beyotime Biotechnology, Shanghai, China). Viral supernatants were collected at 48 h post-transfection, filtered through 0.22 μm membranes, and used to infect A549 cells.

### 2.6 Preparation of anti-GAS5-P50 antibody

The GAS5-P50-specific polyclonal antibody was custom-generated by Zoonbio Biotechnology Co., Ltd. (Nanjing, China). Briefly, two antigenic peptides VLKSKPNSSHW-C and CPQGRMRIAT were chemically synthesized and conjugated to keyhole limpet hemocyanin (KLH) via cysteine-mediated crosslinking. New Zealand White rabbits were immunized with the peptide-KLH conjugate through a standardized protocol involving primary immunization and multiple booster injections. Following titer evaluation by ELISA, the antiserum was affinity-purified using the immunizing peptides.

### 2.7 Western blotting

Cell lysates were prepared and subjected to Western blot analysis as previously described (Wang et al., 2021). Briefly, protein samples were separated by SDS-PAGE and subsequently transferred onto nitrocellulose membranes using a wet electrophoresis system. After blocking with 5% non-fat milk for 1 h, the membranes were incubated with the indicated antibodies. Protein signals were visualized by chemiluminescence detection using an Odyssey XF Imaging System (LI-COR Bioscience).

### 2.8 Hemagglutination (HA) assay and plaque assay

For the HA assay, serially diluted virus samples were mixed with 0.5% chicken erythrocytes in V-bottom 96-well plates and incubated at room temperature for 20 min. The HA titer was determined as the highest dilution showing complete agglutination. For the plaque assay, confluent MDCK cells in 6-well plates were infected with 10-fold serially diluted virus for 1 h, overlaid with α-minimal essential medium containing 1.5% low-melting-point agarose (Promega, WI, USA) and 2 μg/mL TPCK-trypsin (Sigma-Aldrich, MO, USA), and incubated at 37°C for 72 h. Plaques were visualized by crystal violet staining, and viral titers were calculated.

### 2.9 Cell counting kit-8 (CCK-8) assay

Cell viability was assessed using CCK-8 assay according to the manufacturer’s protocol. Briefly, cells were seeded into 96-well plates at a density of 5 × 10^3^ cells per well and incubated overnight. After the experimental treatments, 10 μL of CCK-8 reagent (Beyotime Biotechnology, Shanghai, China) was added to each well, followed by incubation at 37°C for 1 h. Absorbance was measured at 450 nm using a microplate reader (Tecan Infinite M200 Pro, Switzerland). Cell viability was calculated as a percentage relative to untreated controls.

### 2.10 Immunoprecipitation (IP) and mass spectrometry (MS)

Cells were lysed in RIPA buffer supplemented with protease inhibitors, and IP was performed using anti-Flag/HA magnetic beads as previously described (Li et al., 2022). For MS analysis, IP eluates or total protein extracts were digested with trypsin and subsequently analyzed by liquid chromatography-tandem mass spectrometry (LC-MS/MS) at Hoogen Biotech Co. Ltd (Shanghai, China).

### 2.11 Statistical analysis

Student’s *t*-test was used for comparison between two groups. Data are represented as mean ± SD (standard deviation). Differences were considered statistically significant with *p* < 0.05.

## 3 Results

### 3.1 GAS5 is significantly upregulated during IAV infection

**Fig. 1:**
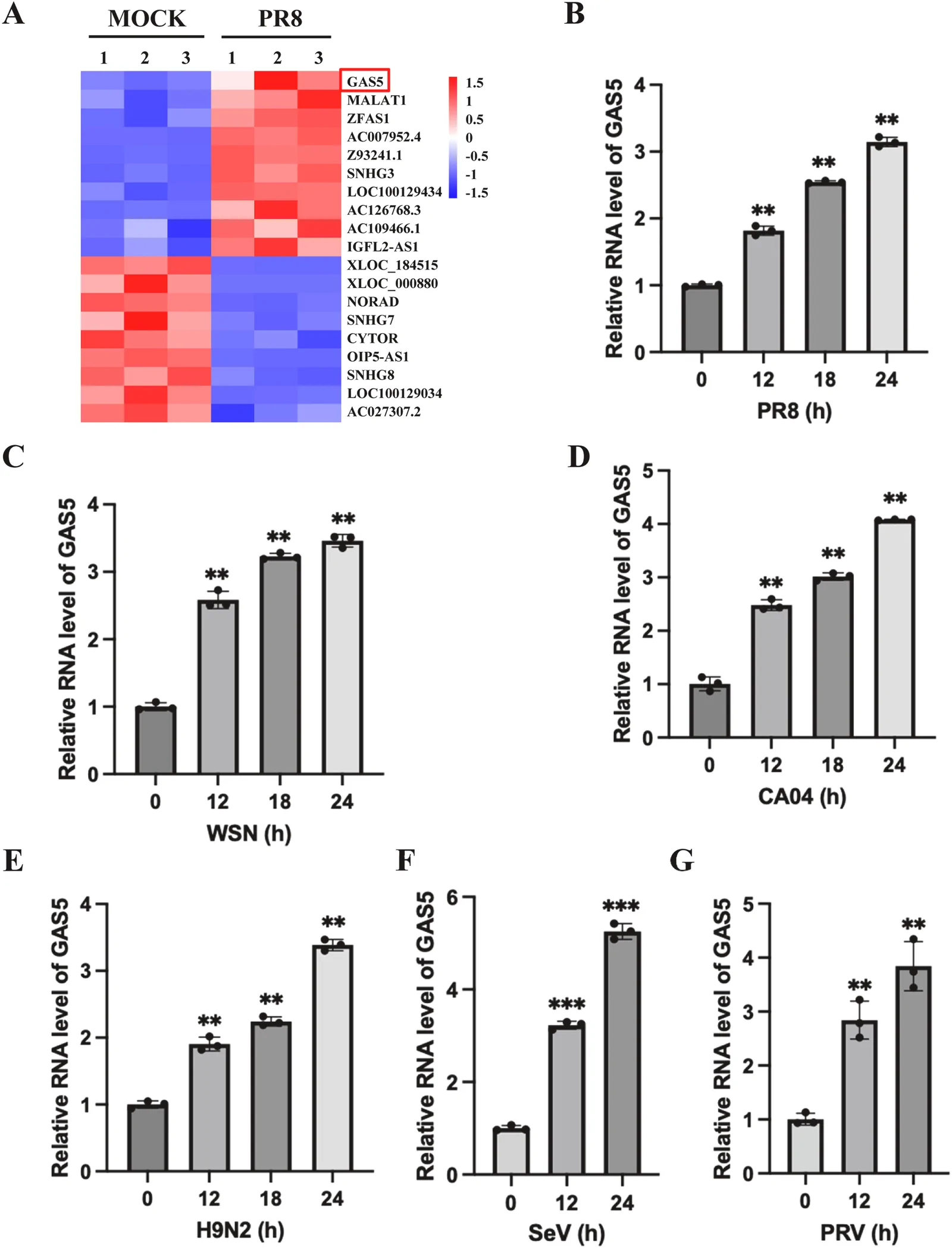
GAS5 is significantly upregulated during IAV infection. (A) RNA-seq analysis of A549 cells infected with the PR8 influenza virus (MOI = 1) for 12 h. Shown are representative lncRNAs whose expressions significantly changed after viral infection. (B-G) A549 cells were infected with PR8 (B), WSN (C), CA04 (D), H9N2 (E), SeV (F), and PRV (G) for the indicated times. Then the expression of GAS5 was examined using RT-qPCR. Data are represented as mean ± SD; Shown are representative data from three biologically independent experiments; **p < 0.01, ***p < 0.001.

Emerging evidence indicates that lncRNAs serve as critical regulators in virus-host interactions. To comprehensively identify lncRNAs involved in influenza virus infection, we conducted RNA sequencing (RNA-seq) analysis of A549 cells infected with IAV compared to uninfected controls (GEO accession: GSE211357). Our transcriptomic profiling revealed GAS5 as one of the most prominently upregulated lncRNAs upon viral infection (Fig. 1A). Subsequent RT-qPCR validation confirmed that GAS5 expression increased progressively in PR8-infected A549 cells over time (Fig. 1B). To further investigate the impact of influenza virus on GAS5 expression, we infected A549 cells with additional H1N1 strains (WSN and CA04) as well as an H9N2 strain and analyzed GAS5 levels at 0, 12, 18, and 24 h post-infection (hpi). Remarkably, all tested influenza virus strains induced significant GAS5 upregulation in a time-dependent manner (Fig. 1C-E).

To determine whether GAS5 induction is specific to influenza virus, we analyzed its expression in response to other viral pathogens. Infection with either Sendai virus (SeV), an RNA virus, or pseudorabies virus (PRV), a DNA virus, led to significant upregulation of GAS5 (Fig. 1F, G). These results indicate that GAS5 induction may reflect a broad, non-specific host response to viral infections.

### 3.2 Type I/III interferons and IL-6 signals mediate GAS5 upregulation

**Fig. 2:**
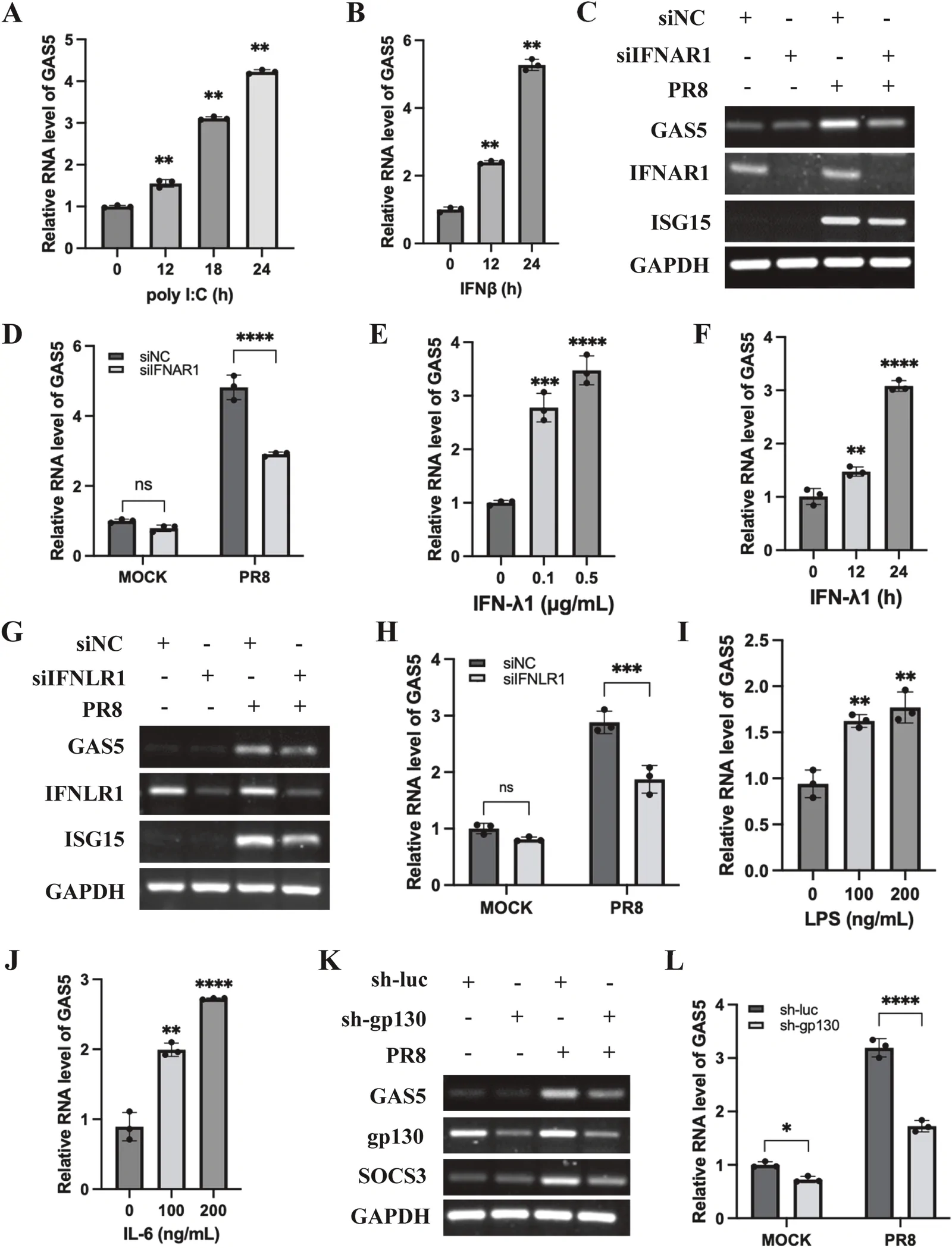
Type I/III interferons or IL-6 treatment induces GAS5 expression. (A, B) A549 cells were stimulated with poly(I:C) (A) or IFN-β (B) for the indicated times. Then GAS5 expression was detected using RT-qPCR. (C, D) A549 cells were transfected with control siRNA or siRNA targeting IFNAR1. After 36 h of transfection, the cells were infected with or without PR8 influenza virus (MOI = 1) for 16 h. Then the expression of IFNAR1 and GAS5 was examined by RT-PCR (C) and RT-qPCR (D). (E, F) A549 cells were stimulated with IFN-λ1 at indicated concentrations (E) or for the indicated times (F). Then GAS5 expression was detected using RT-qPCR. (G, H) A549 cells were transfected with control siRNA or siRNA targeting IFNLR1. After 36 h of transfection, the cells were infected with or without PR8 influenza virus (MOI = 1) for 16 h. Then the expression of IFNLR1 and GAS5 was examined by RT-PCR (G) and RT-qPCR (H). (I, J) A549 cells were stimulated with LPS (I) or IL-6 (J) at indicated concentrations. Then GAS5 expression was detected using RT-qPCR. (K, L) A549 cells stably expressing shRNA targeting gp130 or luciferase control were infected with PR8 influenza virus for 24 h. Then the expression of gp130 and GAS5 was examined by RT-PCR (K) and RT-qPCR (L). Data are represented as mean ± SD; Shown are representative data from three biologically independent experiments; *p < 0.05, **p < 0.01, ***p < 0.001, ****p < 0.0001, and ns represents no significance.

Our findings demonstrate that influenza virus infection induces GAS5 expression. To elucidate the regulatory mechanisms underlying GAS5 induction post-infection, we first examined the effect of poly(I:C), a synthetic double-stranded RNA analog that mimics viral infection. Time-course experiments in A549 cells revealed that poly(I:C) stimulation significantly increased GAS5 expression in a time-dependent manner (Fig. 2A).

Given that poly(I:C) is a well-established potent activator of interferon and inflammatory responses, we first sought to determine whether interferon signaling directly regulates GAS5 expression. To investigate this, we treated A549 cells with type I interferon (IFN-β) and analyzed GAS5 expression at 0, 12, and 24 h post-stimulation. RT-qPCR analysis demonstrated a significant time-dependent upregulation of GAS5 following IFN-β treatment (Fig. 2B). Importantly, siRNA-mediated silencing of the type I IFN receptor IFNAR1 markedly attenuated IAV-induced GAS5 expression compared to control cells (Fig. 2C, D). Similarly, stimulation with type III interferon (IFN-λ1) also induced GAS5 expression in both dose- and time-dependent manners (Fig. 2E, F). Consistent with our observations for type I IFN signaling, siRNA knockdown of the type III IFN receptor IFNLR1 significantly reduced IAV-mediated GAS5 induction relative to controls (Fig. 2G, H). In addition to interferons, we examined the effects of inflammatory mediators on GAS5 expression. Treatment of A549 cells with either LPS or IL-6 resulted in dose-dependent upregulation of GAS5 (Fig. 2I, J). Notably, targeted shRNA knockdown of the IL-6 receptor gp130 substantially diminished IAV-induced GAS5 expression compared to control cells (Fig. 2K, L). Collectively, these results demonstrate that GAS5 expression in A549 cells can be induced by both type I/III interferons and IL-6, highlighting the responsiveness of this lncRNA to diverse cytokine signals.

### 3.3 GAS5 promotes influenza virus replication

**Fig. 3:**
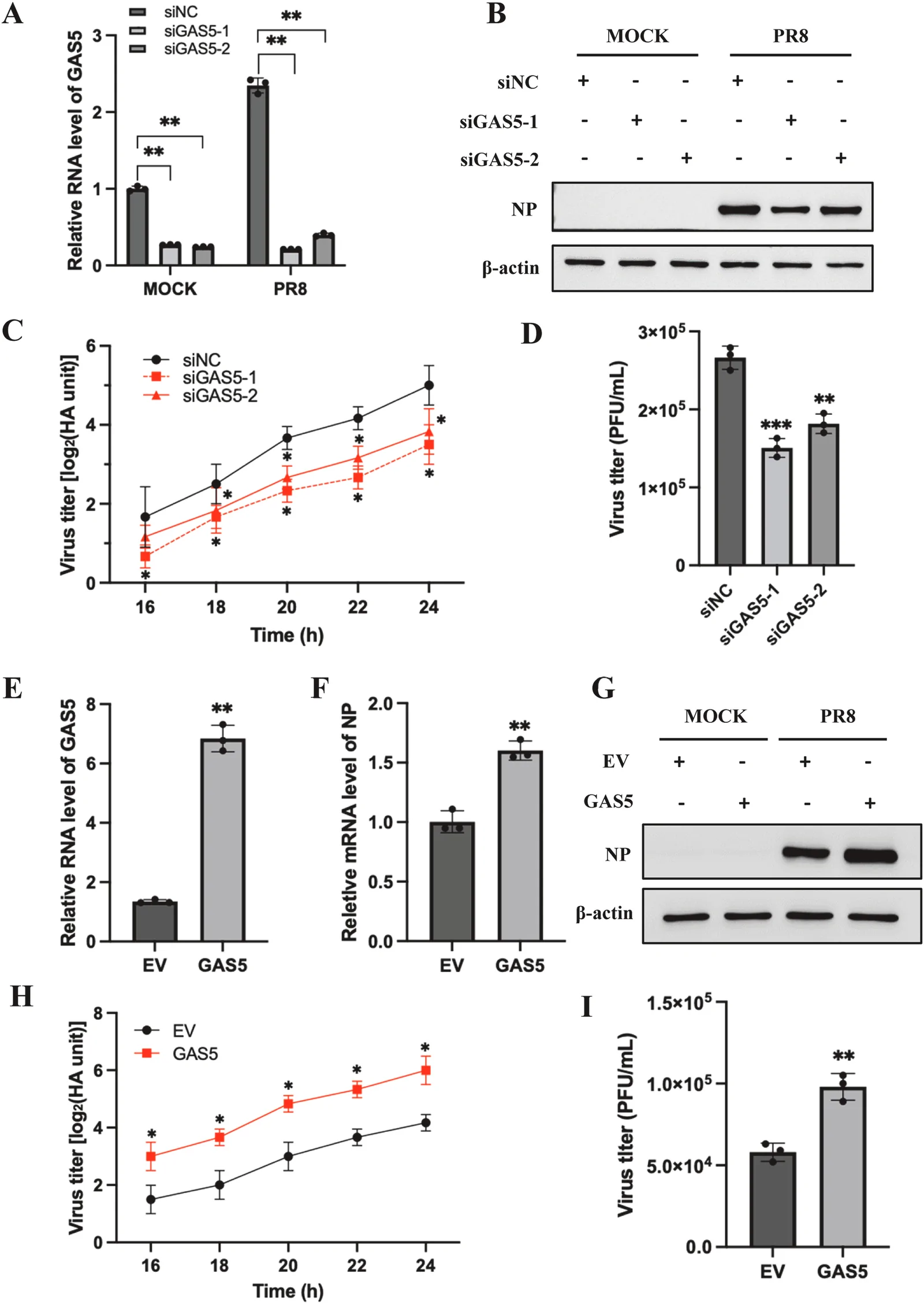
GAS5 promotes influenza virus replication. (A-D) A549 cells were transfected with control siRNA or siRNA targeting GAS5. After 48 h of transfection, the cells were infected with or without PR8 influenza virus. The expression of GAS5 and viral NP expression at 16 hpi was examined by RT-qPCR (A) and Western blotting (B), respectively. The culture supernatants were harvested at the indicated times for HA assay (C) and at 16 hpi for plaque assay (D) to measure virus titers. (E-I) A549 cells stably expressing GAS5 or empty vector (EV) were infected with or without PR8 influenza virus (MOI = 1). The expression of GAS5 and viral NP expression at 16 hpi was examined by RT-qPCR (E, F) and Western blotting (G), respectively. The culture supernatants were harvested at the indicated times for HA assay (H) and at 16 hpi for plaque assay (I) to measure virus titers. Data are represented as mean ± SD; Shown are representative data from three biologically independent experiments; *p < 0.05, **p < 0.01, ***p < 0.001.

To determine whether GAS5 regulates influenza virus replication, we designed two specific siRNAs targeting GAS5. After transfecting A549 cells with either control siRNA or GAS5-targeting siRNAs for 48 h, we infected the cells with PR8 influenza virus. RT-qPCR analysis confirmed efficient GAS5 knockdown both before infection and at 16 hpi (Fig. 3A). Notably, GAS5 silencing significantly reduced viral NP protein expression, as demonstrated by Western blotting (Fig. 3B). Furthermore, hemagglutination (HA) assay of supernatants collected at multiple time points revealed a substantial decrease in viral titers in siRNA-treated cells compared to controls (Fig. 3C). Consistent with these findings, plaque assay confirmed that GAS5 depletion markedly suppressed viral replication (Fig. 3D). Collectively, these results indicate that GAS5 knockdown impairs influenza virus replication.

To further corroborate these observations, we generated a stable GAS5-overexpressing A549 cell line via lentiviral transduction. RT-qPCR analysis confirmed significantly elevated GAS5 levels in these cells following viral infection (Fig. 3E). Correspondingly, NP expression was enhanced in GAS5-overexpressing cells, as measured by both RT-qPCR and Western blotting (Fig. 3F, G). Moreover, HA and plaque assays of supernatants from infected cells demonstrated a pronounced increase in viral titers compared to controls (Fig. 3H, I). Taken together, these data establish that GAS5 overexpression facilitates influenza virus replication.

### 3.4 GAS5 encodes a micropeptide

**Fig. 4:**
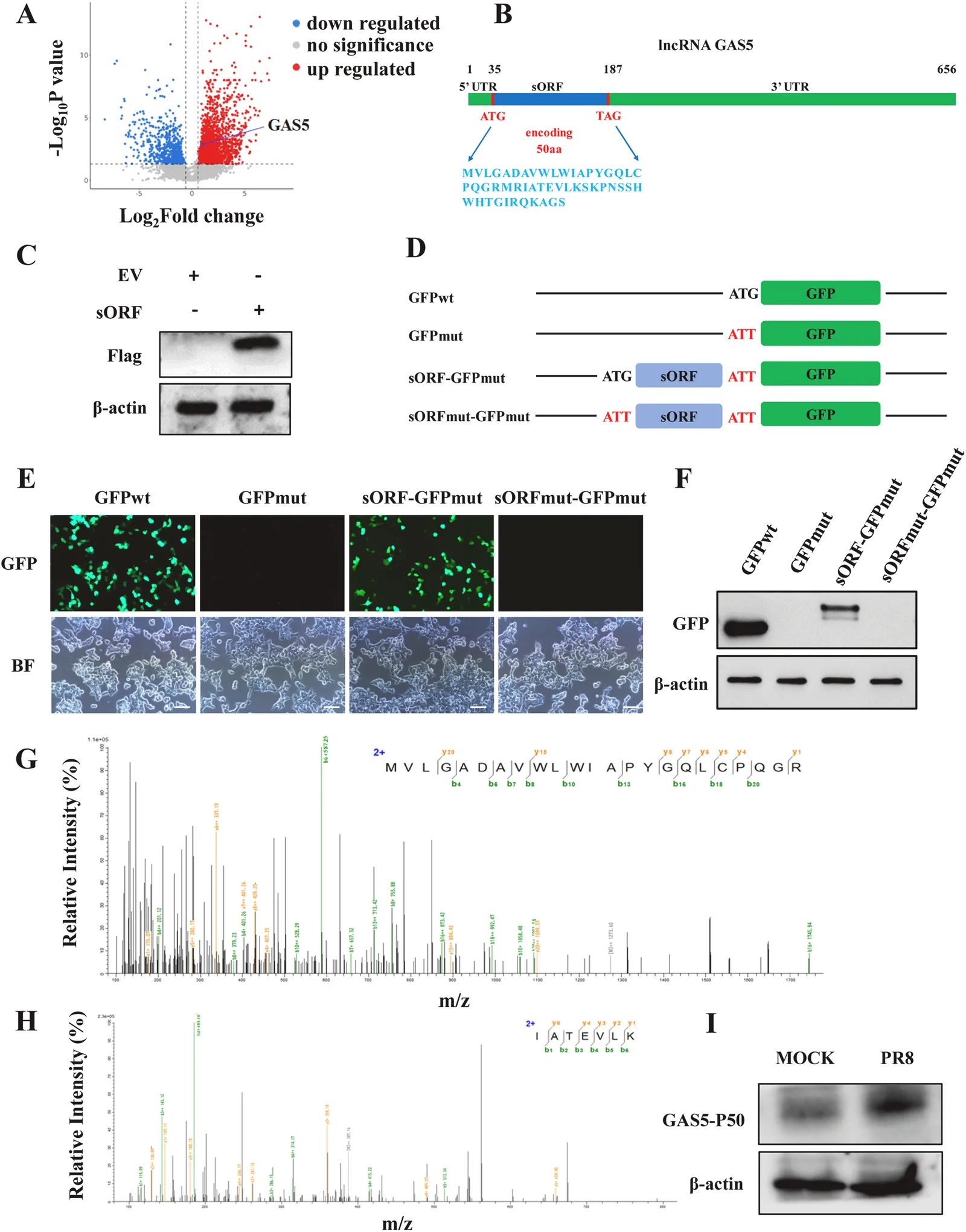
GAS5 promotes influenza virus replication. (A) Volcano plot showing differential translational efficiency (TE) analysis of lncRNAs between PR8-infected cells and control cells. Fold change > 1.5, P value < 0.05. (B) Schematic representation of GAS5 containing a sORF, which may encode a 50-aa micropeptide. (C) The sORF was cloned into pCAGGS vector with a 3 × Flag tag fused to the C-terminus. After transfection into 293T cells for 24 h, expression of the sORF-encoded micropeptide was detected using an anti-Flag antibody. (D) Diagram of the GFP fusion constructs used for transfection. The start codon ATG of the GFP (GFPwt) gene is mutated to ATT (GFPmut), and the sORF was cloned and fused with mutated GFP (sORF-GFPmut). The start codon ATG of the sORF is mutated to ATT (sORFmut-GFPmut). (E, F) The indicated constructs were transfected into 293T cells for 24 h. GFP fluorescence was examined using an inverted fluorescence microscope (E), and protein expression was detected by Western blotting using an anti-GFP antibody (F). Scale bar, 100 μm. (G, H) Mass spectrum of the GAS5-P50 peptide sequence. (I) A549 cells were infected with or without PR8 influenza virus for 24 h. Then the protein was prepared, and the endogenous expression of micropeptide encoded by GAS5 was detected using micropeptide-specific antibody. Shown are representative data from three biologically independent experiments.

Recent studies have demonstrated that certain lncRNAs can encode functional micropeptides, which play critical regulatory roles in diverse cellular processes (Jaunbocus et al., 2025; Xiao et al., 2024). To investigate the coding ability of GAS5, we performed Ribo-seq on A549 cells before and after infection with PR8 influenza virus (GEO accession: GSE252920) and integrated these data with RNA-seq analysis. The results revealed that the translation efficiency (TE) of GAS5 was clearly upregulated upon IAV infection (Fig. 4A), and it contains a small open reading frame (sORF) predicted to encode a 50-amino acid micropeptide (Fig. 4B). We cloned the sORF into a eukaryotic expression vector, generating a construct with a 3 × Flag tag fused to the C-terminus. After transfection into 293T cells, expression of the sORF-encoded micropeptide can be detected using an anti-Flag antibody (Fig. 4C).

To further validate the coding capacity of the GAS5 sORF identified by Ribo-seq, we constructed a series of reporter plasmids based on the pNL-GFP vector. First, we mutated the start codon ATG to ATT in the GFP coding sequence (referred to as GFPmut) to abolish its translational initiation. Next, we inserted the GAS5 sORF (with its stop codon removed) upstream of the mutated GFP sequence, generating the sORF-GFPmut plasmid. As a negative control, we further mutated the start codon of the GAS5 sORF in the sORF-GFPmut plasmid, yielding sORFmut-GFPmut (Fig. 4D). These constructs were transfected into 293T cells, and fluorescence microscopy was performed 24 h post-transfection. Notably, cells transfected with GFPmut exhibited no green fluorescence, whereas those expressing GFPwt and sORF-GFPmut displayed GFP signals. In contrast, fluorescence was abolished in cells transfected with sORFmut-GFPmut (Fig. 4E). To further confirm these findings, we performed Western blotting to detect GFP expression. A GFP fusion protein was detected only in cells transfected with sORF-GFPmut, while neither GFP nor its fusion product was observed in cells expressing GFPmut or sORFmut-GFPmut (Fig. 4F). These findings demonstrate that the GAS5 sORF encodes a micropeptide, which we named GAS5-P50.

To verify the endogenous expression of GAS5-P50, we performed mass spectrometry on protein extracts derived from PR8-infected A549 cells. After SDS-PAGE separation and excision of gel fragments corresponding to regions below 15 kDa, LC-MS/MS analysis revealed two peptide sequences that exactly matched the predicted amino acid sequence of GAS5-P50 (Fig. 4G, H). Additionally, we generated an anti-GAS5-P50 antibody and confirmed the presence and expression of GAS5-P50 in A549 cells by Western blotting (Fig. 4I). Together, these findings provide strong evidence that GAS5-P50 is endogenously expressed in A549 cells upon IAV infection.

### 3.5 GAS5 is dependent on GAS5-P50 to support IAV replication

**Fig. 5:**
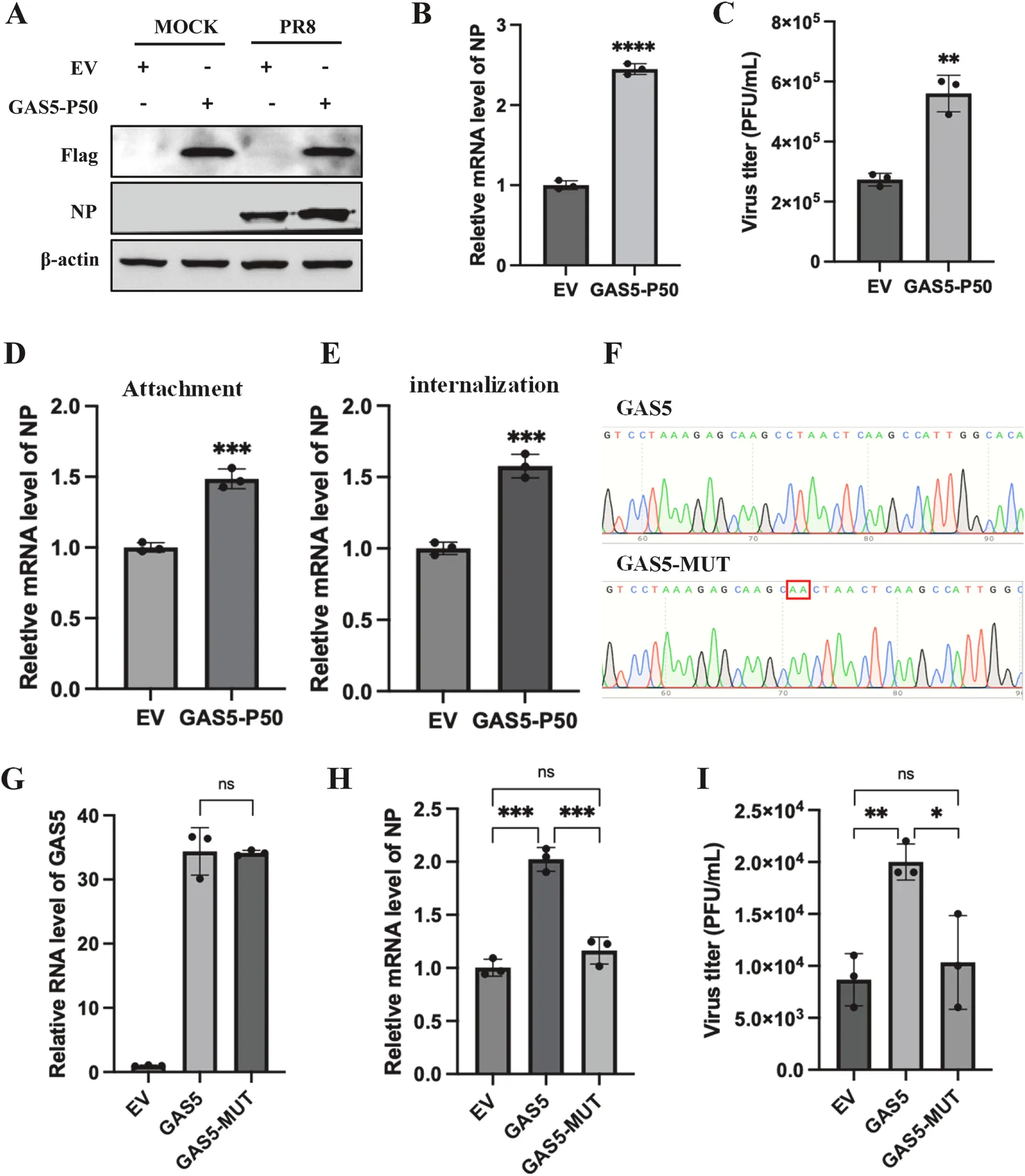
GAS5 requires GAS5-P50 to support IAV replication. (A-C) A549 cells stably expressing GAS5-P50 or empty vector (EV) were infected with or without PR8 influenza virus (MOI = 1). The expression of viral NP expression at 16 hpi was examined by Western blotting (A) and RT-qPCR (B). The culture supernatants were harvested at 16 hpi for plaque assay (C) to measure virus titers. (D) A549 cells stably expressing GAS5-P50 or EV were infected with PR8 influenza virus (MOI = 10) at 4°C for 1 h. After the cells were washed with PBS, RNA was isolated and viral NP was quantified by RT-qPCR. (E) A549 cells stably expressing GAS5-P50 or EV were infected with PR8 influenza virus (MOI = 10) at 4°C for 1 h, and cells were then moved to 37°C for another 1 h. The RNA was isolated and viral NP was quantified by RT-qPCR. (F) A GAS5 mutant plasmid (GAS5-MUT) was constructed by inserting two nucleotides (AA) into the sORF region encoding GAS5-P50 within the full-length GAS5 transcript. The frameshift mutation was verified by sequencing analysis. (G-I) 293T cells were transfected with plasmids expression GAS5, GAS5-MUT, and EV for 16 h, followed by infection with PR8 influenza virus. The expression of GAS5 (G) and viral NP (H) was examined by RT-qPCR. The culture supernatants were harvested at 16 hpi for plaque assay (I) to measure virus titers. Data are represented as mean ± SD; Shown are representative data from three biologically independent experiments; *p < 0.05, **p < 0.01, ***p < 0.001, ****p < 0.0001, and ns represents no significance.

To investigate whether GAS5-P50 affects influenza virus replication, the sORF fragment was cloned into the pLVX3 vector, and a stable cell line overexpressing GAS5-P50 was generated via lentiviral packaging. Both control and GAS5-P50 overexpression cells were infected with PR8 influenza virus. Having confirmed the successful overexpression of GAS5-P50 by Western blotting (Fig. 5A), we observed a concomitant increase in viral NP expression (Fig. 5A, B). Plaque assays of post-infection supernatants revealed that GAS5-P50 overexpression resulted in a significantly higher viral titer than the control (Fig. 5C), indicating that GAS5-P50 promotes influenza virus replication. We next sought to determine which stage of the viral life cycle is influenced by GAS5-P50. A549 cells overexpressing either GAS5-P50 or empty vector (EV) were infected with PR8 virus and incubated at 4°C for 1 hour to allow viral attachment. The cells were then shifted to 37°C and incubated for an additional 1 h to permit viral internalization. Viral RNA levels were quantified by RT-qPCR to evaluate the effects of GAS5-P50 on viral attachment and internalization. The results demonstrated that overexpression of GAS5-P50 in cells facilitates the attachment of influenza virus, leading to a subsequent increase in viral internalization (Fig. 5D, E).

To further elucidate the functional relationship between GAS5 and GAS5-P50, we constructed a GAS5 mutant plasmid (GAS5-MUT) by inserting two nucleotides (AA) into the sORF region encoding GAS5-P50 within the full-length GAS5 transcript. This frameshift mutation disrupts the coding sequence of GAS5-P50. Successful mutagenesis was confirmed by DNA sequencing (Fig. 5F). The plasmids expressing EV, GAS5, and GAS5-MUT were transfected into 293T cells, and RNA samples were subsequently harvested. RT-qPCR analysis revealed significantly increased GAS5 expression levels in cells transfected with GAS5 and GAS5-MUT compared to the control (Fig. 5G). Following influenza virus infection, cells and cell culture supernatants were harvested. Viral NP expression and viral titers were determined using RT-qPCR and plaque assay, respectively. The results demonstrated that both NP expression and viral titers were significantly elevated in GAS5-transfected cells compared to the control. In contrast, cells transfected with GAS5-MUT exhibited a marked reduction in NP expression and viral production relative to the GAS5 group, and showed no significant difference compared to the EV control (Fig. 5H, I). Taken together, these findings indicate that GAS5-P50 plays a critical role in mediating the proviral effect of GAS5 on influenza virus replication.

### 3.6 Synthetic GAS5-P50 promotes IAV replication both *in vitro* and *in vivo*

**Fig. 6:**
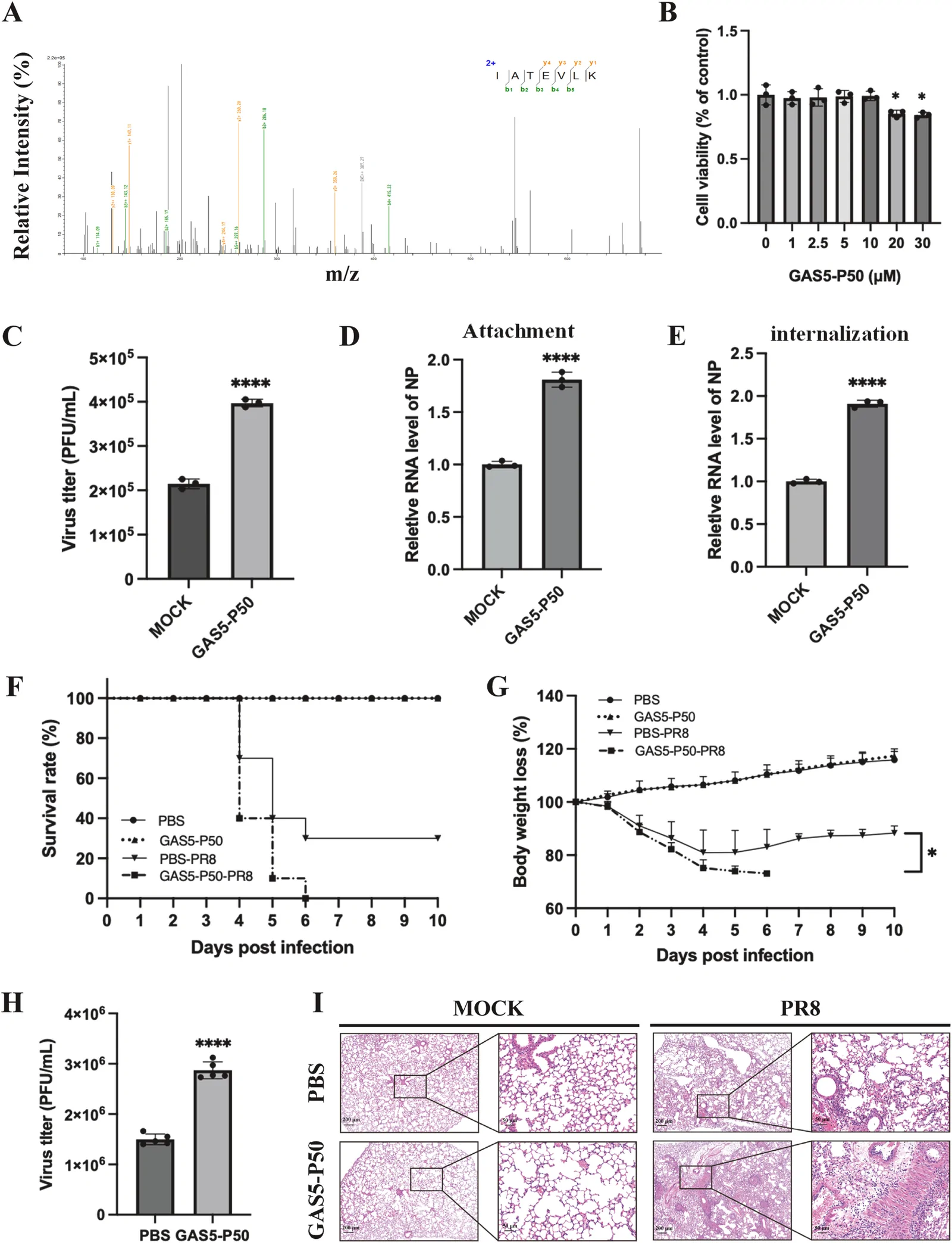
Synthetic GAS5-P50 promotes IAV replication both *in vitro* and *in vivo*. (A) Mass spectrometric detection of the GAS5-p50 peptide in the supernatant of PR8 influenza virus-infected cells. (B) CCK-8 assay was performed to measure the proliferation of A549 cells treated with synthetic GAS5-P50 at the indicated concentrations. (C) A549 cells were treated with or without synthetic GAS5-P50 (10 μM), followed by infection with PR8 influenza virus. The culture supernatants were harvested at 16 hpi for plaque assay to measure virus titers. (D) A549 cells treated with or without synthetic GAS5-P50 (10 μM) were infected with PR8 influenza virus (MOI = 10) at 4°C for 1 h. After the cells were washed with PBS, RNA was isolated and viral NP was quantified by RT-qPCR. (E) A549 cells treated with or without synthetic GAS5-P50 (10 μM) were infected with PR8 influenza virus (MOI = 10) at 4°C for 1 h, and cells were then moved to 37°C for 1 h. The RNA was isolated and viral NP was quantified by RT-qPCR. (F-I) Six- to eight-week-old female C57BL/6 J mice were pretreated intraperitoneally with synthetic GAS5-P50 (5 mg/kg) or control agents, followed by intranasal inoculation with 5 × 10^4^ PFU of PR8 influenza virus. The mice then received daily intraperitoneal injections of synthetic GAS5-P50 or control compounds. The survival rates (*n* = 10 per group) (F), body weight loss (*n* = 10 per group) (G), lung viral loads at 2 days post-infection (dpi) (H), and lung tissue histology assessed by H&E staining at 2 dpi (I) were analyzed. Data are represented as mean ± SD; Shown are representative data from three biologically independent experiments; *p < 0.05, ****p < 0.0001.

Using DeepTMHMM, GAS5-P50 was predicted to be a secreted micropeptide and 1–13 amino acids as its signal peptide (Fig. S1). Mass spectrometry analysis also confirmed the presence of GAS5-P50 in the supernatant of A549 cells upon IAV infection (Fig. 6A). To further investigate the role of GAS5-P50 in IAV infection, we synthesized GAS5-P50 without the signal peptide to examine its effects *in vitro*. The CCK-8 assay showed that synthesized GAS5-P50 did not significantly reduce A549 cell viability at concentrations up to 10 μM, establishing a non-cytotoxic dose range for subsequent experiments. Cytotoxicity was observed at higher concentrations (20 and 30 μM) (Fig. 6B). Then A549 cells were infected with IAV and treated with 10 μM of the synthetic micropeptide. Western blotting showed increased expression of viral PB2 proteins (Fig. S2A). Plaque assay further demonstrated that GAS5-P50 treatment significantly elevated viral titers in the cell supernatant (Fig. 6C). Additionally, it was observed that synthesized GAS5-P50 treatment promoted viral attachment and the subsequent internalization into host cells (Fig. 6D, E). These results are consistent with above findings obtained from overexpression of GAS5-P50 in cells.

To validate the function of synthetic GAS5-P50 *in vivo*, C57BL/6 J mice were intranasally infected with PR8 influenza virus and treated with GAS5-P50 (5 mg/kg) or the PBS control. Administration of GAS5-P50 significantly reduced the survival rate and exacerbated body weight loss in infected mice compared to the control (Fig. 6F, G). Furthermore, mice treated with GAS5-P50 exhibited increased PB2 expression (Fig. S2B, C). Importantly, no significant difference in PB2 expression was observed between PBS- and scrambled peptide-treated infected mice (Fig. S2D), confirming that the proviral effect of GAS5-P50 is sequence-specific and not due to peptide contaminants or non-specific properties. This increase in PB2 was consistent with the observed elevation in lung viral titers (Fig. 6H). Histopathological analysis revealed enhanced inflammatory cell infiltration, alveolar wall thickening, and hemorrhage in GAS5-P50-treated mice (Figs. 6I and S2E). To determine whether this proviral effect is conserved across influenza A virus subtypes, we challenged mice with a swine-origin H3N2 strain. Consistent with the results observed with H1N1 infection, GAS5-P50 treatment in H3N2-infected mice significantly increased lung viral titers and exacerbated histopathological damage (Fig. S2F-H), indicating a broad proviral role for GAS5-P50. Our findings demonstrate that synthetic GAS5-P50 promotes IAV replication *in vitro*. The *in vivo* data further confirm that GAS5-P50 exacerbates disease severity and exhibits a conserved proviral effect across subtypes, highlighting its potential as a broad-spectrum target for antiviral therapy.

### 3.7 GAS5-P50 promotes Wnt/β-catenin signaling via interaction with NOTUM

**Fig. 7:**
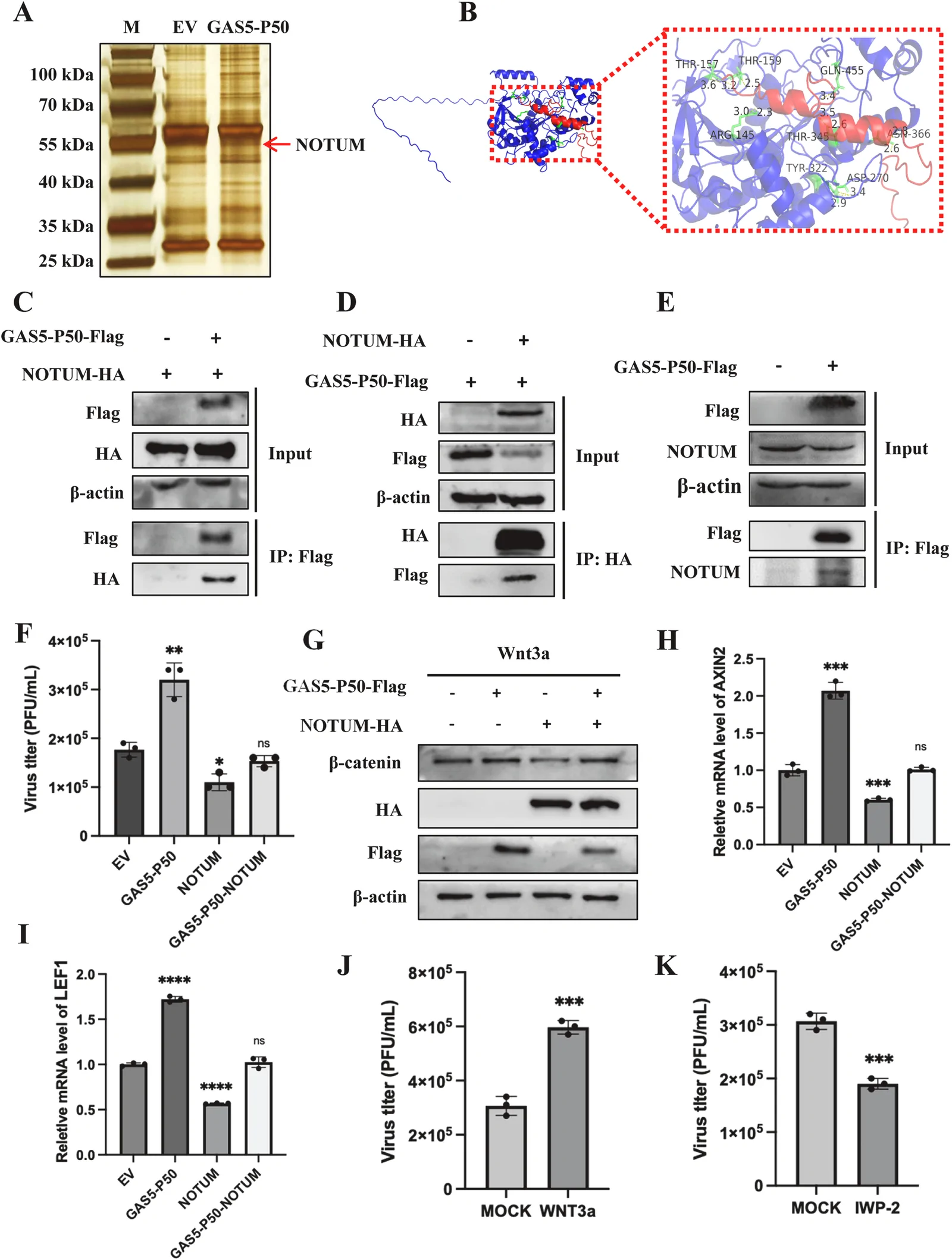
GAS5-P50 interacts with NOTUM to enhance Wnt/β-catenin activation. (A) Proteins interacting with GAS5-P50 were identified through immunoprecipitation (IP) and subsequent silver staining. (B) Molecular docking of GAS5-P50 and NOTUM protein. (C, D) 293T cells were co-transfected with GAS5-P50-Flag and NOTUM-HA plasmids, followed by infection with PR8 influenza virus. GAS5-P50-Flag was immunoprecipitated from the lysates with anti-Flag antibody and the precipitates were blotted with anti-HA antibody (C). NOTUM-HA was immunoprecipitated from the lysates with anti-HA antibody and the precipitates were blotted with anti-Flag antibody (D). (E) 293T cells were transfected with either EV or GAS5-P50-Flag plasmids, followed by infection with PR8 influenza virus. GAS5-P50-Flag was immunoprecipitated from the lysates with anti-Flag antibody and the precipitates were subjected to immunoblotting with anti-NOTUM antibody. (F) 293T cells were transfected with EV, GAS5-P50, NOTUM, or GAS5-P50 together with NOTUM, respectively. After 16 h of infection with PR8 influenza virus, viral titers in the supernatants were determined by plaque assay. (G) 293T cells were transfected with EV, GAS5-P50, NOTUM, or GAS5-P50 together with NOTUM, followed by treatment with Wnt3a (1 μg/mL). The expression of β-catenin was examined by Western blotting. (H, I) 293T cells were transfected with EV, GAS5-P50, NOTUM, or GAS5-P50 together with NOTUM, respectively. After 16 h of infection with PR8 influenza virus, the expression of AXIN2 (H) and LEF1 (I) was examined by RT-qPCR. (J, K) A549 cells were treated with Wnt3a (1 μg/mL) (J) or IWP-2 (50 μM) (K), followed by infection with PR8 influenza virus for 16 h. Viral titers in the supernatants were determined by plaque assay. Data are represented as mean ± SD; Shown are representative data from three biologically independent experiments; *p < 0.05, **p < 0.01, ***p < 0.001, ****p < 0.0001, and ns represents no significance.

Using a combination of immunoprecipitation (IP) and silver staining followed by mass spectrometry, we identified NOTUM as a novel interaction partner of GAS5-P50 (Fig. 7A). Molecular docking analysis performed with AlphaFold 3 predicted a strong interaction between GAS5-P50 and NOTUM, with a binding free energy of -258.96 kcal/mol. Structural visualization and analysis with PyMOL 3.1 revealed the formation of hydrogen bonds between GAS5-P50 and NOTUM, with key interaction residues including R145, T157, T159, D270, Y322, T345, N366, and Q455 (Fig. 7B). The physical interaction was further validated by co-immunoprecipitation (co-IP) assays (Fig. 7C, D). Additionally, IP experiments confirmed that overexpressed GAS5-P50 interacts with endogenous NOTUM (Fig. 7E). Functionally, overexpression of NOTUM significantly suppressed influenza virus replication. However, when NOTUM was co-transfected with GAS5-P50, viral replication was restored to levels similar to those in EV-transfected cells (Figs. 7F and S3A), indicating that GAS5-P50 antagonizes the antiviral activity of NOTUM.

Given that NOTUM is a known inhibitor of the Wnt/β-catenin signaling pathway (Kakugawa et al., 2015), we investigated whether GAS5-P50 modulates this pathway. Western blot analysis revealed that GAS5-P50 elevated the expression of β-catenin, the key effector of canonical Wnt signaling. Notably, the inhibitory effect of NOTUM on β-catenin was effectively counteracted by the co-expression of GAS5-P50, as evidenced by the restoration of β-catenin to levels comparable to the EV control (Figs. 7G and S3B). To further substantiate the functional impact on Wnt/β-catenin signaling, we examined the mRNA levels of established Wnt target genes, AXIN2 and LEF1, by RT-qPCR. Consistent with the β-catenin protein data, overexpression of GAS5-P50 significantly increased AXIN2 and LEF1 transcription. Conversely, NOTUM overexpression markedly suppressed the expression of these target genes. Importantly, the co-expression of GAS5-P50 with NOTUM restored AXIN2 and LEF1 mRNA levels to those comparable with the control group (Fig. 7H, I). This suggests that GAS5-P50 functionally activates Wnt/β-catenin signaling primarily through antagonizing NOTUM-mediated inhibition. Furthermore, activation of Wnt signaling by Wnt3a treatment obviously promoted virus replication, while treatment of cells with a Wnt pathway inhibitor led to a significant reduction in influenza virus replication (Figs. 7J, K, and S3C, D). These results demonstrate that GAS5-P50 interacts with NOTUM to relieve its suppression of the Wnt signaling pathway, thereby promoting influenza virus replication.

## 4 Discussion

Micropeptides, short protein sequences derived from non-coding regions of the genome, have emerged as pivotal regulators in cellular function (Azam et al., 2025). They originate from diverse sources such as lncRNAs, which can encode micropeptides through small open reading frames (sORFs) within their sequences, and circular RNAs (circRNAs), formed via back-splicing events that also contain sORFs (Jaunbocus et al., 2025). Additionally, upstream open reading frames (uORFs) and downstream open reading frames (dORFs) located in the untranslated regions of mRNAs can be translated to yield micropeptides that regulate gene expression and cell signaling (Dever et al., 2020). Alternative splicing can further contribute to the diversity of micropeptides by generating novel sORFs within mRNA transcripts (Couso and Patraquim, 2017). The functions of these micropeptides are multifaceted and include gene regulation, where they may interact with transcription factors or other regulatory proteins; modulation of cell signaling pathways, thereby influencing cellular responses to various stimuli; and participation in protein-protein interactions, potentially disrupting or enhancing complex formations (Couso and Patraquim, 2017; Jaunbocus et al., 2025). They have also been implicated in immunomodulatory functions by targeting key signaling hubs like MAVS and NLRP3 to suppress or activate immune pathways (Sun et al., 2025). Moreover, there is a growing body of evidence linking the dysregulation of micropeptide expression to disease pathogenesis, particularly in pathogen infection (Chi et al., 2024; Shi et al., 2023b; Zhang et al., 2022).

LncRNA GAS5 has been extensively characterized in oncogenesis (Mourtada-Maarabouni et al., 2009; Ni et al., 2019; Pelisenco et al., 2024; Wang et al., 2016; Yang et al., 2021), however, its potential role in viral infections, including influenza virus, remains poorly understood. Our study demonstrates that GAS5 expression is significantly upregulated during IAV infection. This finding aligns with emerging evidence that lncRNAs are dynamically regulated during viral infections and often participate in host defense or viral immune evasion mechanisms (Amir and Taube, 2024; Bamunuarachchi et al., 2021; Liu et al., 2023; Wang and Cen, 2020; Wang, 2018). Notably, this induction appears to be mediated, at least in part, by type I and type III interferons (IFNs) as well as IL-6, since exogenous treatment with these cytokines similarly elevated GAS5 levels. The critical role of IFN and IL-6 signaling was further supported by the observation that knockdown of their respective receptors attenuated the virus-induced upregulation of GAS5. These findings suggest that GAS5 may function as a downstream effector of innate immune pathways during viral infection, potentially serving as a link between cytokine signaling and host antiviral responses. The fact that GAS5 upregulation is blunted upon receptor knockdown implies that its expression is tightly coupled to cytokine receptor activation, possibly through JAK-STAT or other downstream signaling cascades. Further studies are warranted to elucidate the precise transcriptional or post-transcriptional mechanisms by which these pathways regulate GAS5.

A key finding of this study is that GAS5 exerts a proviral function by enhancing IAV replication. While lncRNAs have been implicated in various viral infections, their roles can be either antiviral (Basavappa et al., 2022; Chai et al., 2018) or proviral (Cao et al., 2023; Liu et al., 2020). Our results position GAS5 as a facilitator of IAV replication, suggesting that it may modulate host cell pathways to create a favorable environment for the virus. Notably, we discovered that GAS5 encodes a micropeptide, GAS5-P50. In over-expression settings GAS5-P50 partially recapitulates the GAS5 phenotype; its quantitative contribution at endogenous levels awaits absolute quantification. This finding challenges the conventional view of GAS5 as a purely non-coding RNA and adds to the growing body of evidence that some lncRNAs harbor sORFs encoding functional micropeptides. The fact that synthetic GAS5-P50 alone can promote IAV replication both *in vitro* and *in vivo* underscores its biological significance. This observation raises intriguing questions about whether other lncRNAs previously assumed to be non-coding might similarly encode functional peptides that influence viral infections.

Mechanistically, we identified NOTUM as a binding partner of GAS5-P50. NOTUM is a secreted carboxylesterase known to inhibit Wnt signaling by cleaving palmitoleate moieties from Wnt proteins (Kakugawa et al., 2015; Shukla et al., 2024). Wnt signaling pathway is initiated when a Wnt ligand binds to a cell surface receptor complex composed of a Frizzled (FZD) receptor and a low-density lipoprotein receptor-related protein 5 or 6 (LRP5/6) co-receptor. Upon ligand binding, a series of intracellular events are triggered that ultimately lead to the stabilization and accumulation of β-catenin in the cytoplasm. In the absence of Wnt signaling, β-catenin is typically degraded by a destruction complex that includes adenomatous polyposis coli (APC), axin, and glycogen synthase kinase 3 (GSK3). However, Wnt signaling disrupts this complex, allowing β-catenin to translocate to the nucleus where it interacts with T cell factor/lymphoid enhancer factor (TCF/LEF) transcription factors to activate the transcription of Wnt target genes (Clevers and Nusse, 2012; Liu et al., 2022; Macdonald et al., 2007). Our data demonstrated that GAS5-P50 interacts with NOTUM, potentially interfering with its enzymatic activity and thereby leading to enhanced activation of the Wnt/β-catenin pathway. This model is supported by our observations that GAS5-P50 restores β-catenin protein levels and, crucially, rescues the expression of downstream target genes (AXIN2 and LEF1) from NOTUM-mediated suppression. It remains possible that GAS5-P50 may influence the Wnt/β-catenin pathway through additional, NOTUM-independent mechanisms; however, our data clearly establish its interaction with and functional antagonism of NOTUM as a central mechanism underlying enhanced viral replication. Structural predictions from AlphaFold 3 further suggest specific residues may mediate the GAS5-P50–NOTUM interaction; however, these computational insights remain to be experimentally verified through approaches such as site-directed mutagenesis. Given that Wnt signaling has been implicated in various viral infections, including hepatitis C virus (HCV), Chikungunya virus (CHIKV), porcine reproductive and respiratory syndrome virus (PRRSV), and IAV (Chatterjee et al., 2023; More et al., 2018; Moreau et al., 2015; Wang et al., 2020), our findings provide a plausible mechanism by which GAS5-P50 supports IAV replication. Further studies should explore whether GAS5-P50-mediated Wnt activation affects specific downstream targets that are known to influence viral life cycles.

Moreover, we observed that the protein level of GAS5-P50 was significantly reduced when co-expressed with NOTUM, compared to the empty vector control. This finding suggests that the interaction with NOTUM may not be neutral; instead, it could destabilize GAS5-P50, potentially by promoting its degradation. This observation reveals a layer of dynamic regulation in this host-virus interaction. This phenomenon implies that the interplay between GAS5-P50 and NOTUM is not a simple, static switch but rather a transient interaction that may lead to the turnover of the complex. Such dynamic regulation could be crucial for achieving a precise and timely control over the Wnt signaling pathway, ensuring an effective antiviral response is not perpetually suppressed, which might be detrimental to the host cell.

The discovery that GAS5 and its encoded micropeptide promote IAV replication opens new avenues for antiviral therapy. Targeting GAS5-P50 or its interaction with NOTUM could represent a novel strategy to limit influenza virus propagation. For instance, small-molecule inhibitors or peptide-based antagonists disrupting the GAS5-P50/NOTUM interaction might attenuate Wnt activation and thereby suppress viral replication. Additionally, since GAS5-P50 is a small peptide, it may be amenable to antibody-based neutralization or CRISPR-based knockout approaches.

In summary, our study establishes GAS5 as a critical host factor upregulated during IAV infection in an IFN and IL-6-dependent manner. We reveal that GAS5 promotes viral replication through its encoded micropeptide, GAS5-P50, which interacts with NOTUM to enhance Wnt/β-catenin signaling. These findings not only advance our understanding of lncRNA-mediated regulation in viral infections but also highlight GAS5-P50 as a potential target for antiviral intervention. Future work should further dissect the molecular mechanisms underlying GAS5-P50’s proviral effects and explore its therapeutic applicability in combating influenza and other viral diseases.

## Financial statement

This work was supported by 10.13039/501100012166National Key Research and Development Program of China (2021YFD1800205), 10.13039/501100001809National Natural Science Foundation of China (32573353, U23A20235), Major Science and Technology Program of Fujian Province of China (2024NZ029018), and Science and Technology Innovation Project of 10.13039/501100008766Fujian Agriculture and Forestry University (KFB23094).

## Data availability

All relevant data are within the Manuscript, Figures and Supplemental Materials. The data from RNA-seq and Ribo-seq have been deposited in GEO public database under the accession numbers GSE211357 and GSE252920, respectively.

## Ethics approval

The animal protocol used in this study was approved by the Research Ethics Committee of Fujian Agriculture and Forestry University (Permit Number PZCASFAFU24037). All animal experiments were performed in accordance with the Regulations for the Administration of Affairs Concerning Experimental Animals of the Ministry of Science and Technology of the People’s Republic of China.

## CRediT authorship contribution statement

**Xinni Zhou:** Investigation, Data curation, Formal analysis. **Xiaojuan Chi:** Data curation, Investigation, Writing – original draft. **Benqun Peng:** Investigation, Data curation. **Ming Gao:** Investigation. **Ning Li:** Investigation. **Lu Liu:** Investigation. **Jie Zeng:** Investigation. **Yuxin Li:** Formal analysis. **Yuzhang Chen:** Formal analysis. **Song Wang:** Conceptualization, Funding acquisition, Project administration, Supervision, Writing – review & editing.

## Declaration of competing interest

The authors declare that they have no known competing financial interests or personal relationships that could have appeared to influence the work reported in this paper.

## Appendix Supplementary materials

Image, application 1
