# Supplementary material for: The lncRNA GAS5-encoded micropeptide facilitates influenza virus replication through modulation of the Wnt/β-catenin signaling pathway
Source: Curr Res Microb Sci. 2026 Jan 27;10:100559. doi: 10.1016/j.crmicr.2026.100559 (PMC12874442; doi:10.1016/j.crmicr.2026.100559)
Supplement: Supplementary file 1 [file mmc1.pdf]

## Fig. S1

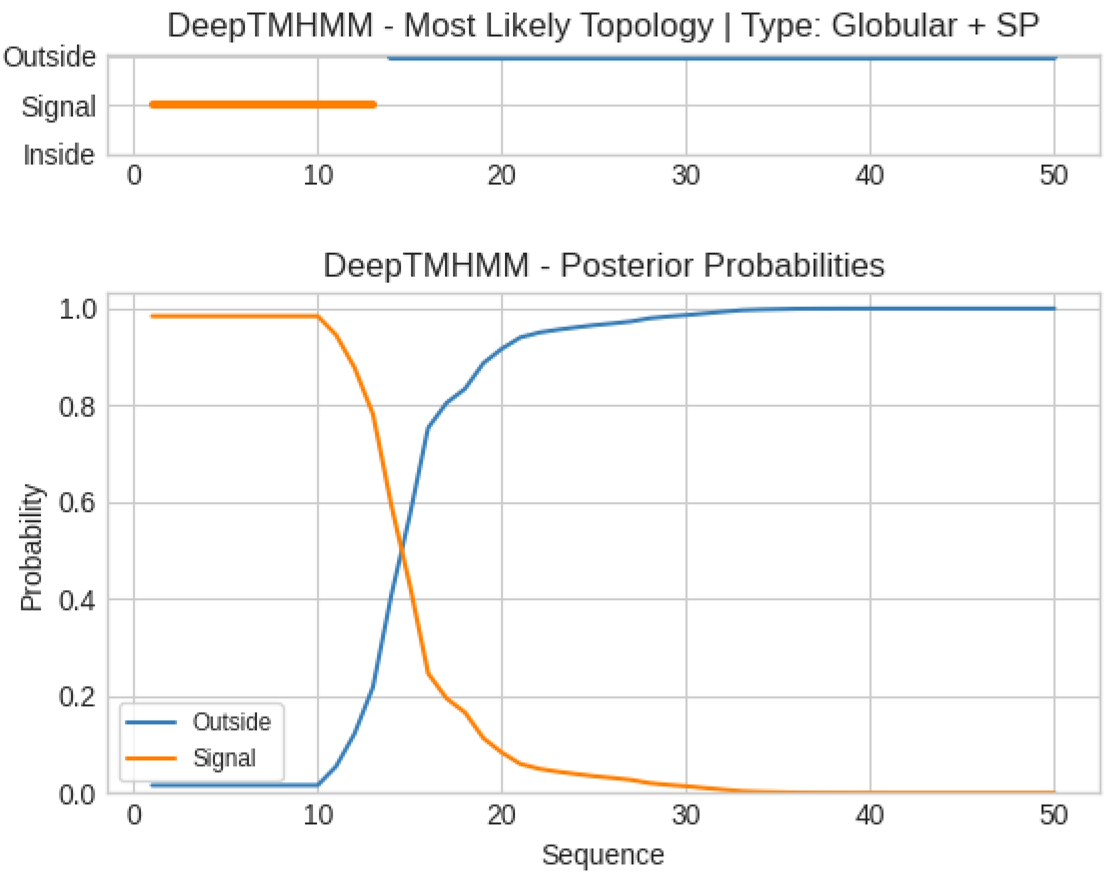


### Predicted Topologies

```
>GAS5-P50 | SP
MVLGADAVWLWIAPYGQLCPQGRMRIATEVLKSKPNSSHWHTGIRQKAGS
SSSSSSSSSSSSSOOOOOOOOOOOOOOOOOOOOOOOOOOOOOOOOOOOOO
```

```
##gff-version 3
# GAS5-P50 Length: 50
# GAS5-P50 Number of predicted TMRs: 0
GAS5-P50 signal 1 13
GAS5-P50 outside 14 50
```

**Fig. S1** Subcellular localization prediction of GAS5-P50 was performed using DeepTMHMM-1.0 (https://services.healthtech.dtu.dk/services/DeepTMHMM-1.0/).

## Fig. S2

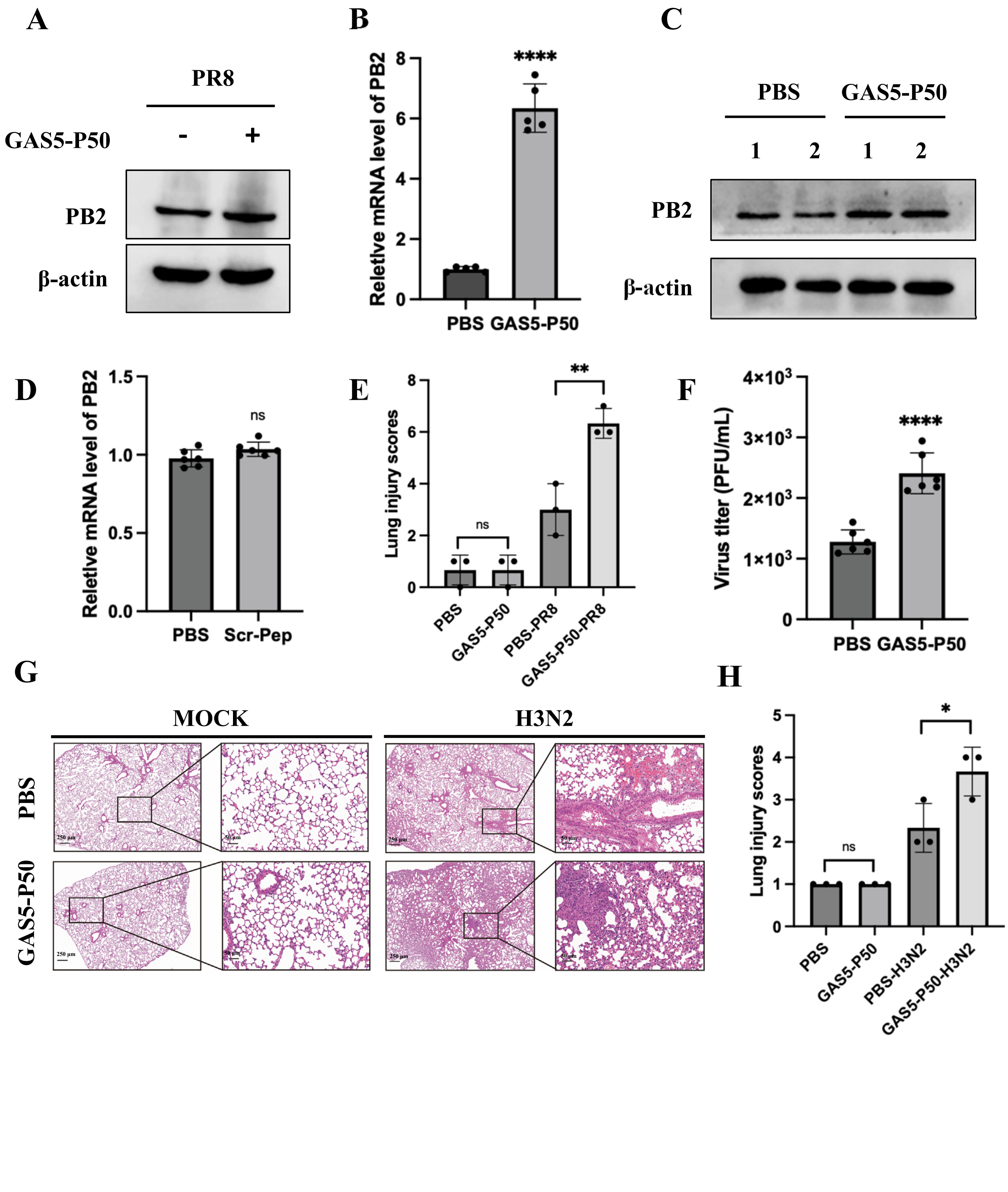
A
PR8
GAS5-P50
-
+
PB2
β-actin
B
****
Reletive mRNA level of PB2
8
6
4
2
0
PBS
GAS5-P50
C
PBS
GAS5-P50
1
2
1
2
PB2
β-actin
D
ns
Reletive mRNA level of PB2
1.5
1.0
0.5
0.0
PBS
Scr-Pep
E
**
ns
Lung injury scores
8
6
4
2
0
PBS
GAS5-P50
PBS-PR8
GAS5-P50-PR8
F
****
Virus titer (PFU/mL)
4×10³
3×10³
2×10³
1×10³
0
PBS
GAS5-P50
G
MOCK
H3N2
PBS
GAS5-P50
H
*
ns
Lung injury scores
5
4
3
2
1
0
PBS
GAS5-P50
PBS-H3N2
GAS5-P50-H3N2

**Fig. S2 Synthetic GAS5-P50 promotes IAV replication both *in vitro* and *in vivo*.** (A) A549 cells were treated with or without synthetic GAS5-P50 (10 μM), followed by infection with PR8 influenza virus. The expression of viral PB2 expression at 16 hours post-infection (hpi) was examined by Western blotting. (B, C) Six- to eight-week-old C57BL/6J mice (n=5 per group) were pretreated intraperitoneally with synthetic GAS5-P50 (5 mg/kg) or control agents, followed by inoculation with $5\times10^4$ PFU of PR8 influenza virus. The mice then received daily intraperitoneal injections of synthetic GAS5-P50 or control compounds. Viral PB2 expression in lung tissues at 2 days post-infection (dpi) was analyzed by RT-qPCR (B) and Western blotting (C). (D) Six- to eight-week-old C57BL/6J mice (n=5 per group) were pretreated intraperitoneally with PBS or synthetic scrambled peptide (Scr-Pep, 5 mg/kg) with an identical composition to GAS5-P50, followed by inoculation with $5\times10^4$ PFU of PR8 influenza virus. The mice then received daily intraperitoneal injections of PBS or the scrambled peptide. Viral PB2 expression in lung tissues at 2 dpi was analyzed by RT-qPCR. (E) Blinded histopathological scores for lung sections from virus-infected mice in Fig. 6I. Pathology was evaluated using a 0-4 scale assessing perivascular cuffing, alveolar exudate, and epithelial damage. (F-H) Six- to eight-week-old C57BL/6J female mice were pretreated intraperitoneally with synthetic GAS5-P50 (5 mg/kg) or control agents, followed by inoculation with $5\times10^4$ PFU of H3N2 influenza virus. Daily intraperitoneal injections of GAS5-P50 or PBS control were subsequently administered. At 2 dpi, viral loads in mouse lungs (F), lung tissue histology by H&E staining (G), and corresponding blinded histopathological scores (H) were assessed. Data are represented as mean ± SD; Shown are representative data from three biologically independent experiments; *$p < 0.05$, **$p < 0.01$, ****$p < 0.0001$, and ns represents no significance.

**Fig. S3**

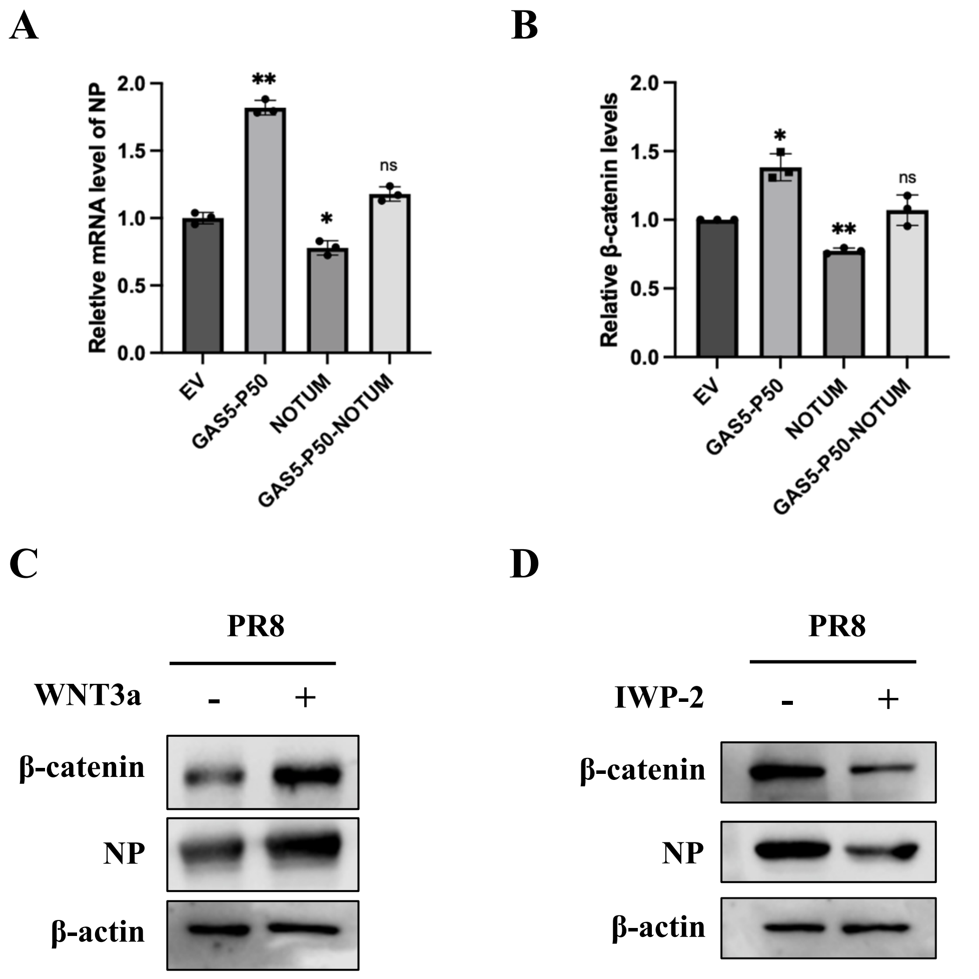


**Fig. S3 GAS5-P50 regulates Wnt/β-catenin activation through interacting with NOTUM.** (A) 293T cells were transfected with EV, GAS5-P50, NOTUM, or GAS5-P50 together with NOTUM, respectively. After 16 h of infection with PR8 influenza virus, viral NP expression was measured by RT-qPCR. (B) β-catenin protein levels in Fig. 7G were quantitated by densitometry and normalized to β-actin levels. (C, D) A549 cells were treated with Wnt3a (1 μg/mL) (C) or IWP-2 (50 μM) (D), followed by infection with PR8 influenza virus for 16 h. The protein expression of indicated genes was examined by Western blotting. Data are represented as mean ± SD; Shown are representative data from three biologically independent experiments; *$p < 0.05$, **$p < 0.01$, and ns represents no significance.

**Table S1 The sequence of primers, siRNA, and shRNA used in the study**

| primers, siRNA and shRNA | Sequence |
|---|---|
| GAS5-F | 5’-AAGGACATGAAGACAGTTCCTG-3’ |
| GAS5-R | 5’-CTGCATGCTTGCTTGTTGTG-3’ |
| GAS5-P50-F | 5’-ATGGTGCTGGGTGCAGATGC-3’ |
| GAS5-P50-R | 5’-ACTTCCAGCTTTCTGTCTAA-3’ |
| IFNAR1-F | 5’-CGTACAAGCATCTGATGG-3’ |
| IFNAR1-R | 5’-GCATTTGAAGTGTTTTCCC-3’ |
| IFNLR1-F | 5’-ACAACAAGTTCAAGGGACG-3’ |
| IFNLR1-R | 5’-GGAGTGACTGGAAATAGGG-3’ |
| gp130-F | 5’-CAGTGGTCACCTCACACTCC-3’ |
| gp130-R | 5’-GTAGATCTTCTGGCCGCTCC-3’ |
| ISG15-F | 5’-GACCTGACGGTGAAGATGCTG-3’ |
| ISG15-R | 5’-TGCTGCGGCCCTTGTTAT-3’ |
| SOCS3-F | 5’-GACGGAGACTTCGATTCGGG-3’ |
| SOCS3-R | 5’-GCTGGTACTCGCTCTTGGAG-3’ |
| NOTUM-F | 5’-TGAAGGCGTACTCGTTCTTC-3’ |
| NOTUM-R | 5’-CGACTCCAGATACGACACCA-3’ |
| AXIN2-F | 5’-CAACACCAGGCGGAACGAA-3’ |
| AXIN2-R | 5’-GCCCAATAAGGAGTGTAAGGACT-3’ |
| LEF1-F | 5’-AGAACACCCCGATGACGGA-3’ |
| LEF1-R | 5’-GGCATCATTATGTACCCGGAAT-3’ |
| GAPDH-F | 5’-GAGAAGGCTGGGGCTCATTT-3’ |
| GAPDH-R | 5’-AGTGATGGCATGGACTGTGG-3’ |
| Mouse-β-actin-F | 5’-CATTGCTGACAGGATGCAGAAGG-3’ |
| Mouse-β-actin-R | 5’-TGCTGGAAGGTGGACAGTGAGG-3’ |
| IAV NP-F | 5’-CAACCATTATGGCAGCATT-3’ |
| IAV NP-R | 5’-TACTCCTCTGCATTGTCTCC-3’ |
| IAV PB2-F | 5’-ATCTAATGTCGCAGTCTCG-3’ |
| IAV PB2-R | 5’-TTATTGGTCCATTCCTATTC-3’ |
| siGAS5-1 | 5’-GGCUCUGGAUAGCACCUUA-3’ |
| siGAS5-2 | 5’-GGAUGAGAAUAGCUACUGA-3’ |

| | |
|---|---|
| siIFNAR1 | 5’-GCCAAGAUUCAGGAAAUUAUU-3’ |
| siIFNLR1 | 5’-GGGUGGAGUCCGAAUACCUGGAUUA-3’ |
| sh-gp130 | 5’-GCTCACTTGCAACATTCTTAC-3’ |
